# Pharmacokinetics and stability of methoxyflavones from *Kaempferia parviflora* in Thai native roosters

**DOI:** 10.3389/fvets.2025.1582200

**Published:** 2025-04-08

**Authors:** Vibuntita Chankitisakul, Supakorn Authaida, Wuttigrai Boonkum, Sarunya Tuntiyasawasdikul

**Affiliations:** ^1^Department of Animal Science, Faculty of Agriculture, Khon Kaen University, Khon Kaen, Thailand; ^2^Network Center for Animal Breeding and Omics Research, Khon Kaen University, Khon Kaen, Thailand; ^3^Center for Research and Development of Herbal Health Products and Faculty of Pharmaceutical Sciences, Khon Kaen University, Khon Kaen, Thailand

**Keywords:** methoxyflavones, *Kaempferia parviflora*, pharmacokinetics, poultry, stability

## Abstract

**Introduction:**

The present study aimed to characterize the pharmacokinetics and stability of methoxyflavones derived from *Kaempferia parviflora* (KP) in Thai native roosters after oral administration of a KP ethanolic extract.

**Method:**

Twenty-seven male roosters were randomly divided into three groups and received KP extract at different doses of 100, 150, and 200 mg/kg of body weight. Plasma samples were prepared using acetonitrile, ethyl acetate, and a mixture of both to compare the optimal extraction efficiency. Plasma methoxyflavones concentrations were quantified using a validated HPLC method. Pharmacokinetic parameters were calculated using PKSolver. A seven-day stability study assessed methoxyflavones degradation in blood and plasma samples stored at −20°C.

**Results and discussion:**

The results showed that methoxyflavones were rapidly absorbed, reaching maximum plasma concentrations (C_max_) ranging from 0.34 to 0.83 µg/mL within 1.17 to 1.83 hours, with a clear dose-dependent relationship. Elimination was slow, with half-lives ranging from 2.03 to 2.60 hours. The study also found that acetonitrile was the most effective solvent for extracting methoxyflavones from blood samples, yielding recovery rates of 73.95%, 81.49%, and 77.5% for 3,5,7,3′,4′-pentamethoxyflavone (PMF), 5,7-dimethoxyflavone (DMF), and 5,7,4′-trimethoxyflavone (TMF), respectively. High stability was observed in blood and plasma over two days (96.6–100%), with significant degradation (84.3–92.6%) after seven days. This study’s results provide valuable insights for optimizing KP extract use as a poultry feed additive by informing appropriate dosage, extraction, and storage procedures to preserve methoxyflavones integrity.

## 1 Introduction

The poultry industry plays a vital role in global food security, supplying animal-based proteins globally (1). In Thailand, native roosters are commonly raised in open-house systems, where environmental factors may influence the chickens’ reproductive performance, growth, and overall well-being (2). To mitigate these challenges, medicinal herbs have been historically used to promote animal health. In poultry production, herbal additives are increasingly recognized for their potential to enhance overall well-being, prevent diseases, and improve productivity (3). Among these, flavonoids, a group of secondary metabolites found in many plant species, have shown promising benefits for poultry health (4). These bioactive compounds have been reported to enhance meat and egg quality, strengthen immunity, and improve overall rooster health (5–8).

**Figure 1:**
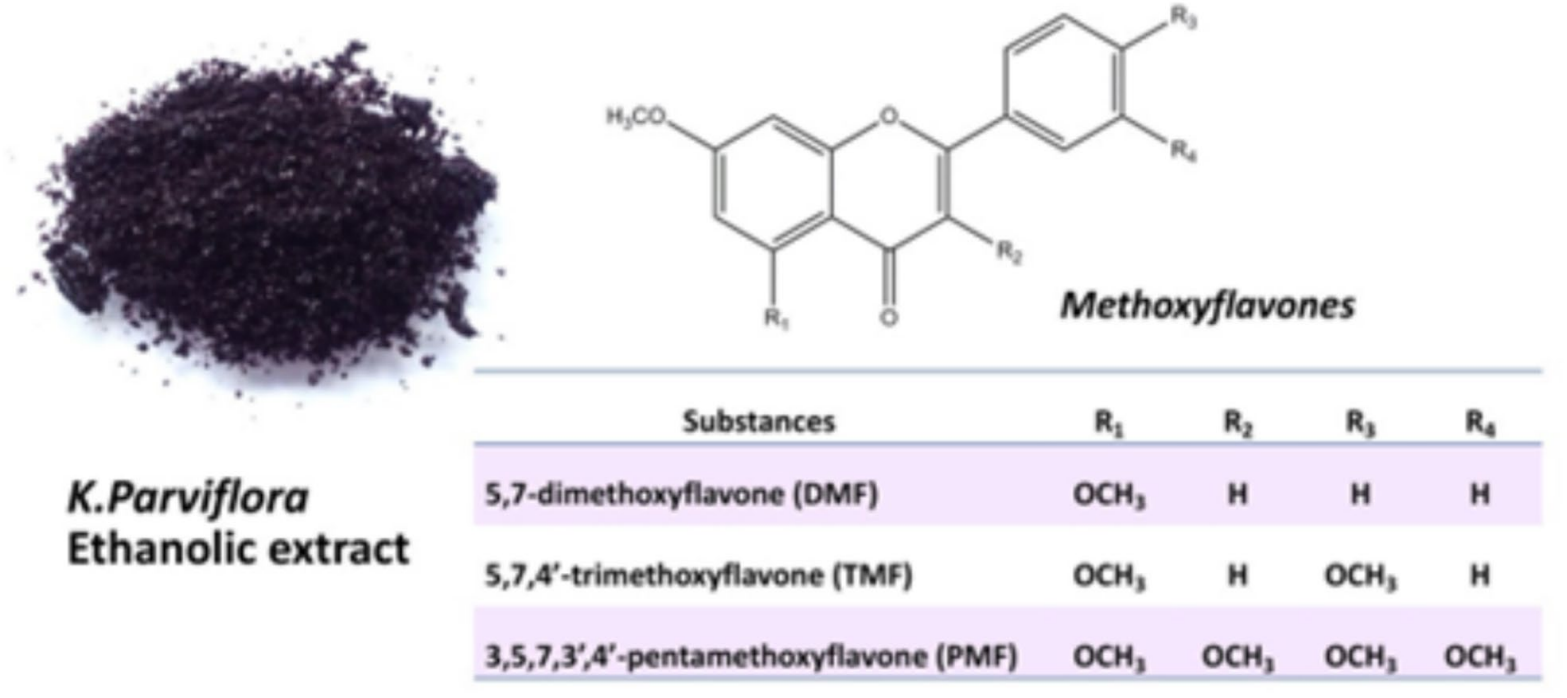
The major chemical structures of methoxyflavones (DMF, TMF, and PMF) are derived from an ethanolic extract of *Kaempferia parviflora*.

One particularly promising flavonoid-rich plant is *Kaempferia parviflora* (KP), commonly known as “Black Ginseng” or “Krachai Dum” in Thailand. This native herb is rich in methoxyflavones, including 5,7-dimethoxyflavone (DMF), 5,7,4′-trimethoxyflavone (TMF), and 3,5,7,3′,4′-pentamethoxyflavone (PMF), which are the primary flavonoid compounds derived from KP extracts (9), as shown in Figure 1. Several studies have shown that ethanolic extracts from KP rhizomes exhibit anti-inflammatory properties, such as inhibiting prostaglandin E2 release and suppressing the mRNA expression of inducible nitric oxide synthase (iNOS) and cyclooxygenase 2 (COX2) in a dose-dependent manner (10). Furthermore, KP has been found to enhance blood circulation, improve endothelial cell function (11), and even support male sexual dysfunction by stimulating spermatogenesis (12, 13). Notably, oral administration of KP extract has been shown to enhance sperm production and maintain rooster semen for extended periods (14).

Despite these benefits, the pharmacokinetics and stability of KP methoxyflavones in poultry remain unclear. Limited research exists on their absorption, distribution, metabolism, and elimination, as well as inconsistencies in dosage recommendations due to variations in molecular structure. Furthermore, high plasma enzyme activity in poultry may accelerate compound degradation (15), while factors like storage temperature and light exposure influence stability, affecting pharmacokinetic accuracy (16, 17).

Therefore, this study aims to characterize the pharmacokinetics and stability of methoxyflavones derived from KP extract in Thai native roosters after oral administration of a KP ethanolic extract. Specifically, it focuses on identifying effective extraction methods for methoxyflavones from blood samples, characterizing their pharmacokinetic parameters and half-life, and assessing the effects of different storage conditions on their stability. The findings will provide valuable insights for developing safe and effective strategies to improve poultry health, potentially enhancing productivity and profitability for the Thai poultry industry.

## 2 Materials and methods

The experimental protocols involving animals were reviewed and approved by the Animal Ethics Committee of Khon Kaen University, Khon Kaen, Thailand (approval numbers. IACUC-KKU-24/65; Reference No. 660201.2.11/121).

### 2.1 Chemicals and reagents

KP rhizomes were obtained from the Loei province of Thailand. The specimens were certified by a botanist and its herbarium voucher specimen (HB 205/67) was located at the Center for Research and Development of Herbal Health Products, Khon Kaen University, Thailand. Standards for methoxyflavones, including 98% DMF and 97% TMF, were obtained from Indofine Chemical Company Inc. (NJ, USA), and the standard for PMF was purified from KP ethanolic extract using column chromatography as described previously (9).

Acetonitrile (LabScan, Bangkok, Thailand), formic acid (Thermo Fisher Scientific, Leicester, UK), heparin (Leo Pharmaceutical Products, Ballerup, Denmark), polyethylene glycol 400 (PEG 400; S. Tong Chemical Co., Ltd., Bangkok, Thailand), and propylene glycol (Namsiang Co., Ltd., Bangkok, Thailand) were used in this study. All other chemicals were of analytical grade.

### 2.2 Preparation of KP Ethanolic extract

The ethanolic extract of KP rhizomes was prepared by modification from Tuntiyasawasdikul et al. (18). Briefly, KP powder was macerated in 95% ethanol in a stainless-steel tank for 3 days. The extract was obtained by rotary evaporation, yielding a 4.75% extract.

### 2.3 Methoxyflavone quantification by HPLC

Methoxyflavones (PMF, DMF, and TMF) were quantified using an Agilent 1,200 series reverse-phase HPLC system, as previously described by Tuntiyasawasdikul and Sripanidkulchai (19). The validated method employed an Agilent Hypersil ODS C18 column (3.5 μm, 4.6 × 150 mm) at 30°*C. mobile* phases consisted of (A) 0.5% acetic acid in acetonitrile and (B) 0.5% acetic acid in deionized water. A gradient elution (5:95 A:B to 60:40 A:B over the run) was used at a flow rate of 1.2 mL/min, with detection at 254 nm. The injection volume was 20 μL.

### 2.4 HPLC method validation

The analytical method was validated for linearity, accuracy, precision, limit of detection (LOD), and limit of quantitation (LOQ) in accordance with the International Conference on Harmonization (ICH) guidelines (20).

#### 2.4.1 Linearity, sensitivity, and limits of quantification

A six-point calibration curve was plotted, revealing the relationship between the peak area ratios (y) of each methoxyflavone and the concentrations of methoxyflavones (x). The slope, intercept, and correlation coefficient regression parameters were calculated and represented as the coefficient of determination (R^2^). The limits of detection (LOD) and quantification (LOQ) were determined for concentrations exhibiting signal-to-noise (S/N) ratios of 3 and 10, respectively.

#### 2.4.2 Accuracy

The accuracy was assessed as the percentage recovery of the added known analyte in the sample relative to the actual value and the relative standard deviation (%RSD). Three concentrations (5, 50, and 100 μg/mL) of standard methoxyflavone mixtures were added into the KP extract sample. Spiked samples were made in triplicate and subjected to three tests. The percentage recovery was calculated using the formula: recovery = (amount detected / amount spiked) × 100. The acceptance requirement for accuracy was established at ≤2% RSD.

#### 2.4.3 Precision

Intra- and inter-day precision were assessed utilizing low-, medium-, and high-quality control samples (LQC, MQC, HQC) at multiple concentrations (5, 50, and 100 μg/mL), expressed as % relative standard deviation (RSD). The quality control sample was analyzed in five replicated samples for intra-day precision and evaluated over five consecutive days for inter-day precision. The acceptance criterion for both intra- and inter-day precision was set at ≤2% RSD.

### 2.5 *In vivo* pharmacokinetic study

#### 2.5.1 Animal selection and husbandry

Twenty-seven male Thai native Pradu Hang Dum breed roosters were selected for this study. The sample size was calculated by using the G*power program with the following parameters: *α* = 0.05, power of test = 0.8, standard deviation = 0.05, and effect size = 0.6. The sample size was nine in each group. The roosters, aged 36 weeks, exhibited a body weight ranging from 2.8 to 3.0 kg. All animals were obtained from the Research and Development Network Center of Animal Breeding and Omics at Khon Kaen University, Thailand. The roosters were housed in separate cages (60 × 45 × 45 cm) within an open-house system. They were fed a daily commercial diet of 130 g, containing 16% crude protein. Water was provided ad libitum throughout the study.

The temperature and humidity index (THI) was calculated according to the National Oceanic and Atmospheric Administration (1976): THI = (1.8 × temp +32) – (0.55–0.0055 × RH) × (1.8 × temp – 26), where temp is the temperature (°C), and RH is the relative humidity (%). The average temperature was 27.54°C, and the relative humidity was 72.36%. The THI was 77.98 during the conduct of the experiment.

#### 2.5.2 Blood sampling and processing

Blood samples (1 mL) were collected from the brachial vein of each rooster after gentle restraint. The collected blood was immediately transferred into a 1.5 mL microtube containing 10% heparin and gently inverted several times to ensure thorough mixing and prevent clot formation.

For plasma samples, the plasma was separated by centrifugation at 2,200 × g for 15 min at room temperature.

#### 2.5.3 Optimization of blood extraction

Standardized extraction methods are crucial to determine the presence of active compounds in blood samples accurately. The blood samples were collected from the roosters and pooled before undergoing extraction. A known concentration of KP extract was added to 200 μL aliquots of the pooled rooster blood. Two organic solvents, acetonitrile and ethyl acetate, were selected due to their ability to dissolve the lipophilic compounds and generate precipitated protein. Three different extraction methods were compared: Method 1 (M1) involved the addition of 1 mL of acetonitrile, Method 2 (M2) involved the addition of 1 mL of ethyl acetate, and Method 3 (M3) involved the addition of a mixture of acetonitrile and ethyl acetate. After adding the solvent, each sample was vortexed for 2 min and sonicated for 5 min. Subsequently, the samples were centrifuged at 2,000 × g for 2 min. The resulting supernatant was then transferred to clean microtubes, evaporated to dryness, and then reconstituted with 200 μL of methanol. Finally, the samples were filtered through 0.45-μm syringe filters and analyzed using an HPLC assay. Each extraction method was replicated three times.

#### 2.5.4 Pharmacokinetics analysis

The roosters were randomly divided into three groups (*n* = 9 per group) and orally administered KP extract at different doses: 100, 150, and 200 mg/kg of body weight. The KP extract was prepared by dissolving in a solution of 28% propylene glycol, 35% polyethylene glycol 400, 2% ethanol, and deionized water (added until the total volume was 100%). For the method of animal administration, two individuals administered the KP extract to the chickens. One person restrained each chicken by holding its wings and legs, while the other administered the extract orally via a 10 mL syringe, according to body weight.

After oral administration, blood samples were collected at certain time intervals (0.5, 1, 2, 4, 6, and 12 h). The samples were then stored at −20°C until further analysis.

To extract methoxyflavones from the blood samples, all samples were extracted by liquid–liquid extraction two times using acetonitrile. Each sample was vortexed for 2 min and sonicated for 5 min with 1 mL of acetonitrile. After centrifugation at 2,000 × g for 2 min, the supernatant was transferred to clean microtubes, evaporated to dryness, and reconstituted with 200 μL of methanol. The samples were then filtered through 0.45-μm syringe filters and analyzed using an HPLC assay. Pharmacokinetic parameters were determined using the PKSolver add-in program (21).

### 2.6 Methoxyflavone stability in blood and plasma

To assess the stability of methoxyflavones in biological samples, we investigated the degradation of PMF, DMF, and TMF in rooster blood and plasma, a short-term stability study was conducted over a period of 7 days. As previous studies have shown high levels of plasma enzyme activity in rats (15), potentially leading to the breakdown of methoxyflavones, this evaluation was crucial for determining the appropriate handling and storage of samples.

Freshly collected blood and plasma samples were used as controls, while quality control samples were prepared by spiking drug-free rooster blood and plasma with KP extract. All samples were stored at −20°C. The stability of methoxyflavones was assessed at 0, 1, 2, 3, and 7 days of storage. The extraction process described in the previous section was then performed on each sample to quantify the degradation of methoxyflavones. This experimental design included six replicates for each time point.

### 2.7 Statistical analysis

Statistical analysis was conducted using SPSS version 28.0. A one-way ANOVA with a completely randomized design was used to compare the treatment means. Tukey’s *post-hoc* test was applied to determine significant group differences (*p* < 0.05). All results were expressed as mean ± SD.

## 3 Results

### 3.1 HPLC method validation and methoxyflavones quantification

**Figure 2:**
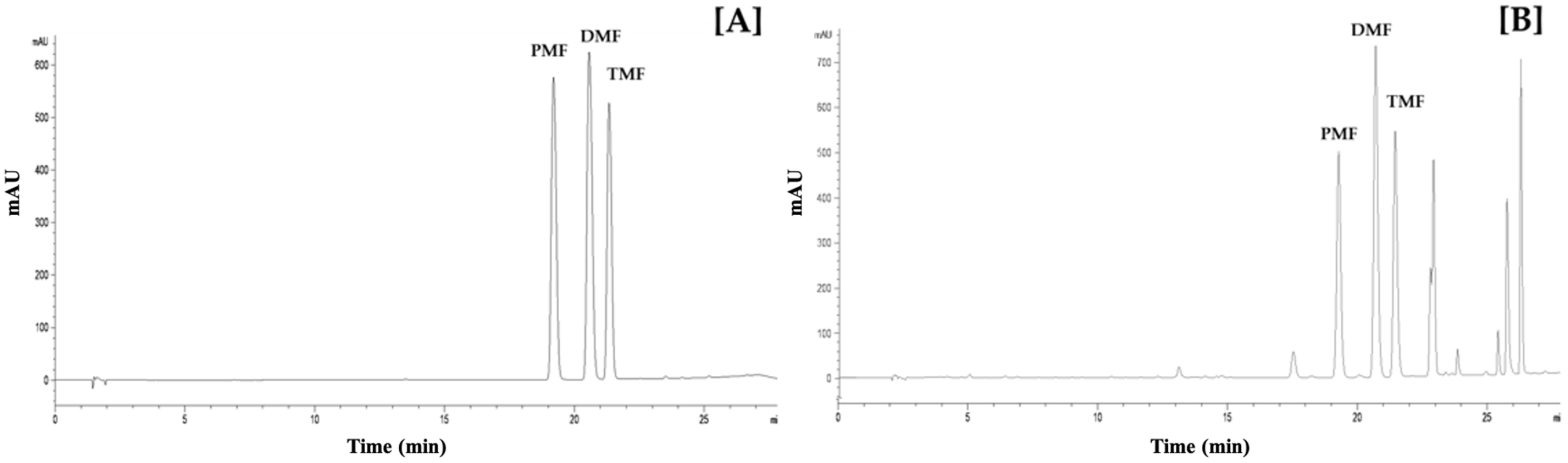
HPLC chromatogram of standard methoxyflavones **(A)** and KP extract **(B)**.

The HPLC method demonstrated excellent linearity (R^2^ > 0.9996, 1–100 μg/mL range), indicating a good linearity of the method (R2 > 0.999). The method’s LOD and LOQ ranged from 0.70 to 1.01 μg/mL and 2.12 to 3.07 μg/mL, respectively (Table 1). Figure 2 displays representative HPLC chromatograms, which show retention times of 19.3, 20.7, and 21.4 min for PMF, DMF, and TMF, respectively. The precision (intra-day and inter-day) demonstrated excellent precision with high repeatability and reproducibility for all three concentrations tested (5, 50, and 100 μg/mL), as indicated by %RSD values lower than 2% (Table 2). In addition, the accuracy for all three methoxyflavones (PMF, DMF, TMF) at concentrations of 5, 50, and 100 μg/mL revealed percentage recoveries ranging from 99.59 ± 0.49 to 104.38 ± 0.21%, which fall within the acceptable range of 95–105%. The %RSD for each concentration level was also less than 2% (Table 3). The quantified amounts of PMF, DMF, and TMF in the KP extract were 25.19 ± 0.94, 22.94 ± 1.29, and 42.54 ± 0.81 mg/g, respectively.

**Table 1:** Quantitative parameters for determination of methoxyflavones.

Parameter	PMF	DMF	TMF
Linearity range (μg/ml)	1–100	1–100	1–100
Regression equation	y = 70.76x – 68.55^a^	y = 81.26x – 78.75^a^	y = 59.82x – 62.27^a^
Linearity (R^2^)	0.9996	0.9996	0.9986
LOD (μg/ml)	1.01 ± 0.05	0.70 ± 0.02	0.85 ± 0.04
LOQ (μg/ml)	3.07 ± 0.15	2.12 ± 0.06	2.56 ± 0.11

a y = ax + b, where y is peak area, x is the concentration of the analyzed sample.

**Table 2:** Validation of precision of the HPLC method for methoxyflavones.

Methoxyflavones	Theoretical concentration (μg/mL)	Intra-day^a^ (*n* = 3)		Inter-day^b^ (*n* = 15)	
		Measured concentration (μg/mL)	RSD (%)^c^	Measured concentration (μg/mL)	RSD (%)^c^
PMF	5	5.75 ± 0.08	1.32	5.75 ± 0.11	1.96
	50	53.39 ± 0.92	1.71	53.56 ± 0.56	1.04
	100	106.20 ± 1.72	1.62	108.06 ± 1.24	1.15
DMF	5	5.74 ± 0.04	0.69	5.59 ± 0.11	1.90
	50	54.23 ± 0.68	1.26	54.39 ± 0.70	1.28
	100	106.09 ± 1.88	1.77	104.70 ± 1.02	0.97
TMF	5	5.78 ± 0.05	0.92	5.68 ± 0.11	1.97
	50	53.90 ± 0.53	0.98	53.34 ± 1.01	1.89
	100	105.11 ± 0.97	0.92	106.64 ± 2.05	1.92

a All values are mean ± SD as obtained by triplicate analyses in a day.

b All values are mean ± SD, obtained by triplicate analyses per day over 5 days.

c The relative standard deviation (RSD) = SD/mean × 100%.

**Table 3:** Validation of the accuracy of the HPLC method for methoxyflavones.

Methoxyflavones	Spiked volume (μg/ml)	Recovery (%)^a^			Mean ± %RSD
		N1	N2	N3	
PMF	5	104.62	104.23	104.28	104.38 ± 0.21
	50	100.32	99.19	99.60	99.70 ± 0.47
	100	103.24	102.65	102.81	102.90 ± 0.24
DMF	5	100.45	100.85	99.96	100.42 ± 0.36
	50	100.36	99.19	99.51	99.69 ± 0.49
	100	103.40	102.88	103.22	103.17 ± 0.21
TMF	5	100.62	101.16	101.93	101.24 ± 0.53
	50	100.24	99.08	99.43	99.59 ± 0.49
	100	104.26	104.06	102.42	103.58 ± 0.80

a All values are mean as obtained by triplicate analyses in a day.

### 3.2 *In vivo* oral absorption and stability of methoxyflavones in roosters

#### 3.2.1 Blood extraction optimization

The recovery percentages of three methoxyflavones (PMF, DMF, and TMF) were compared for each solvent, as shown in Table 4. Acetonitrile (M1) yielded the highest recovery percentages for all three methoxyflavones: 73.95% for PMF, 81.49% for DMF, and 77.5% for TMF. The acetonitrile and ethyl acetate (M3) mixture showed intermediate recovery rates, while ethyl acetate (M2) resulted in the lowest recovery percentages.

**Table 4:** Recovery percentages of methoxyflavones (PMF, DMF, and TMF) using different extraction solvents from rooster blood.

Methods	%Recovery		
	PMF	DMF	TMF
M1	73.95 ± 1.02 ^a^	81.49 ± 0.94 ^a^	77.50 ± 0.65 ^a^
M2	57.41 ± 0.92 ^c^	66.45 ± 1.08 ^c^	61.55 ± 1.21 ^c^
M3	70.55 ± 0.51 ^b^	73.46 ± 0.36 ^b^	75.76 ± 0.63 ^b^

M1: acetonitrile, M2: ethyl acetate, and M3: a mixture of acetonitrile and ethyl acetate. Different superscript letters (a, b, c) within the same row indicate significant differences at *p* < 0.05. The results are presented as mean ± SD.

#### 3.2.2 Pharmacokinetics

**Figure 3:**
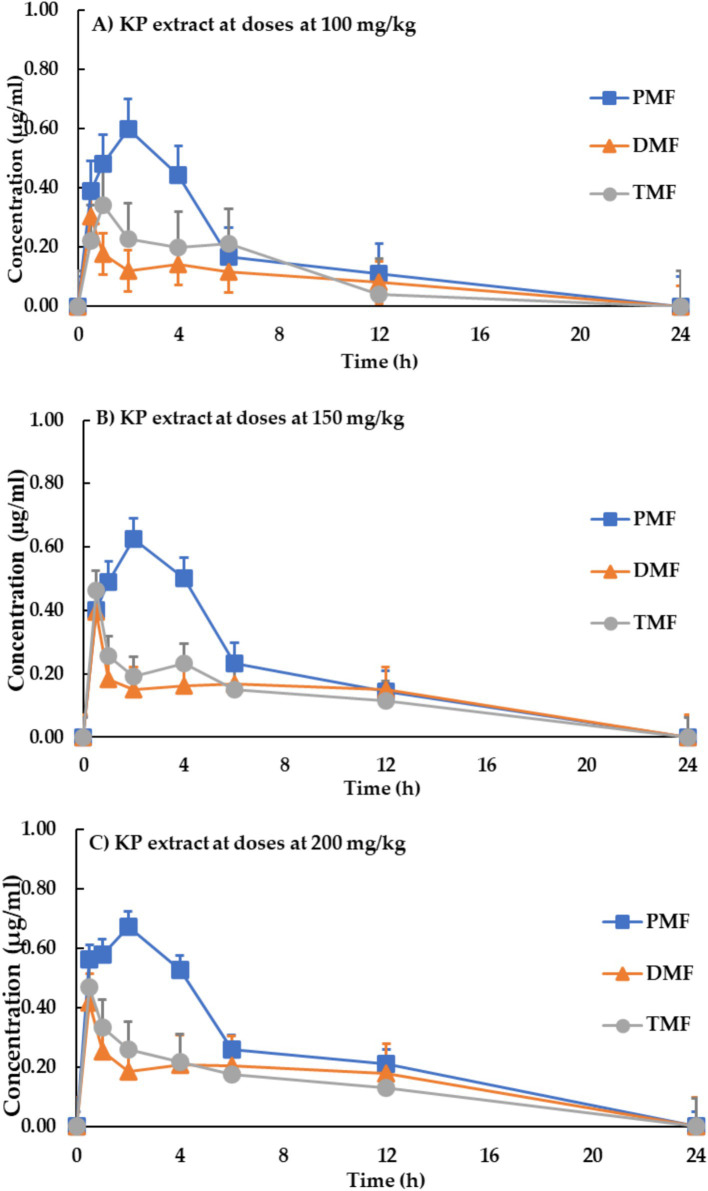
Blood concentration-time profile of methoxyflavones after a single oral administration of KP extract at doses of **(A)** 100 mg/kg, **(B)** 150 mg/kg, and **(C)** 200 mg/kg of body weight. Each data point represents the mean ± SD, *n* = 9.

Pharmacokinetic parameters are presented in Table 5. Meanwhile, Figure 3 shows the blood concentration-time profiles of the three methoxyflavones (PMF, DMF, and TMF) after a single oral administration of KP extract at doses of 100, 150, and 200 mg/kg of body weight.

**Table 5:** Pharmacokinetic parameters of methoxyflavones after a single oral of KP extract in roosters.

Parameters	Treatment								
	KP 100 mg/kg			KP 150 mg/kg			KP 200 mg/kg		
	PMF	DMF	TMF	PMF	DMF	TMF	PMF	DMF	TMF
AUC (h·μg/mL)	2.62 ± 0.34^b^	1.02 ± 0.23^j^	1.20 ± 0.15^y^	3.01 ± 0.42^a,b^	1.45 ± 0.17^i^	1.36 ± 0.15^x^	3.06 ± 0.57^a^	1.68 ± 0.57^i^	1.45 ± 0.12^x^
T_1/2_ (h)	2.03 ± 0.34	2.56 ± 0.49	2.15 ± 0.71	2.24 ± 0.71	2.57 ± 0.20	2.01 ± 0.31	2.27 ± 0.93	2.60 ± 0.28	2.03 ± 0.37
T_max_ (h)	1.83 ± 0.37	1.17 ± 0.37	1.25 ± 0.56	1.80 ± 0.40	1.33 ± 0.49	1.25 ± 0.40	1.83 ± 0.37	1.17 ± 0.37	1.33 ± 0.47
C_max_ (μg/mL)	0.64 ± 0.06^b^	0.34 ± 0.10^j^	0.38 ± 0.09^y^	0.81 ± 0.12^ab^	0.50 ± 0.05^i^	0.53 ± 0.06^x^	0.83 ± 0.11^a^	0.54 ± 0.08^i^	0.59 ± 0.06^x^
*Vd* (mg/kg)	7.78 ± 0.63	18.85 ± 1.78	17.79 ± 5.61	8.24 ± 1.14	19.11 ± 1.78	19.97 ± 3.32	8.69 ± 1.25	19.52 ± 2.28	12.66 ± 1.73
*Cl* (mg/h/kg)	2.46 ± 0.42	8.24 ± 1.78	4.58 ± 0.59	2.83 ± 0.76	7.21 ± 1.66	5.84 ± 0.67	3.22 ± 0.52	7.37 ± 2.21	4.95 ± 1.58
K_e_ (h^−1^)	0.35 ± 0.06	0.28 ± 0.07	0.30 ± 0.08	0.40 ± 0.09	0.30 ± 0.08	0.34 ± 0.03	0.41 ± 0.04	0.33 ± 0.03	0.37 ± 0.03

Different superscript letters (a,b) indicate significant differences between PMF treatments at *p* < 0.05.

Different superscript letters (i,j) indicate significant differences between DMF treatments at *p* < 0.05.

Different superscript letters (x,y) indicate significant differences between TMF treatments at *p* < 0.05.

Oral absorption of all three methoxyflavones was rapid, with maximum concentrations (C_max_) reached within 1.17–1.83 h. C_max_ values ranged from 0.34 to 0.83 μg/mL and exhibited a clear dose-dependent increase. The area under the curve (AUC) values, representing the total drug exposure, also showed a dose-dependent increase, ranging from 2.62 to 3.06 μg·h/mL for PMF, 1.02–1.68 μg·h/mL for DMF, and 1.20–1.45 μg·h/mL for TMF.

The volumes of distribution (Vd) for PMF, DMF, and TMF ranged from 7.78 to 19.52 mg/kg. Eliminating methoxyflavones was relatively slow, with half-lives (T_1/2_) ranging from 2.01 to 2.60 h. The elimination rate constant (Ke) ranged from 0.28 to 0.41 h^−1^, and the clearance value ranged from 2.46 to 8.24 mg/h/kg.

#### 3.2.3 Stability of methoxyflavones in rooter blood and plasma

**Figure 4:**
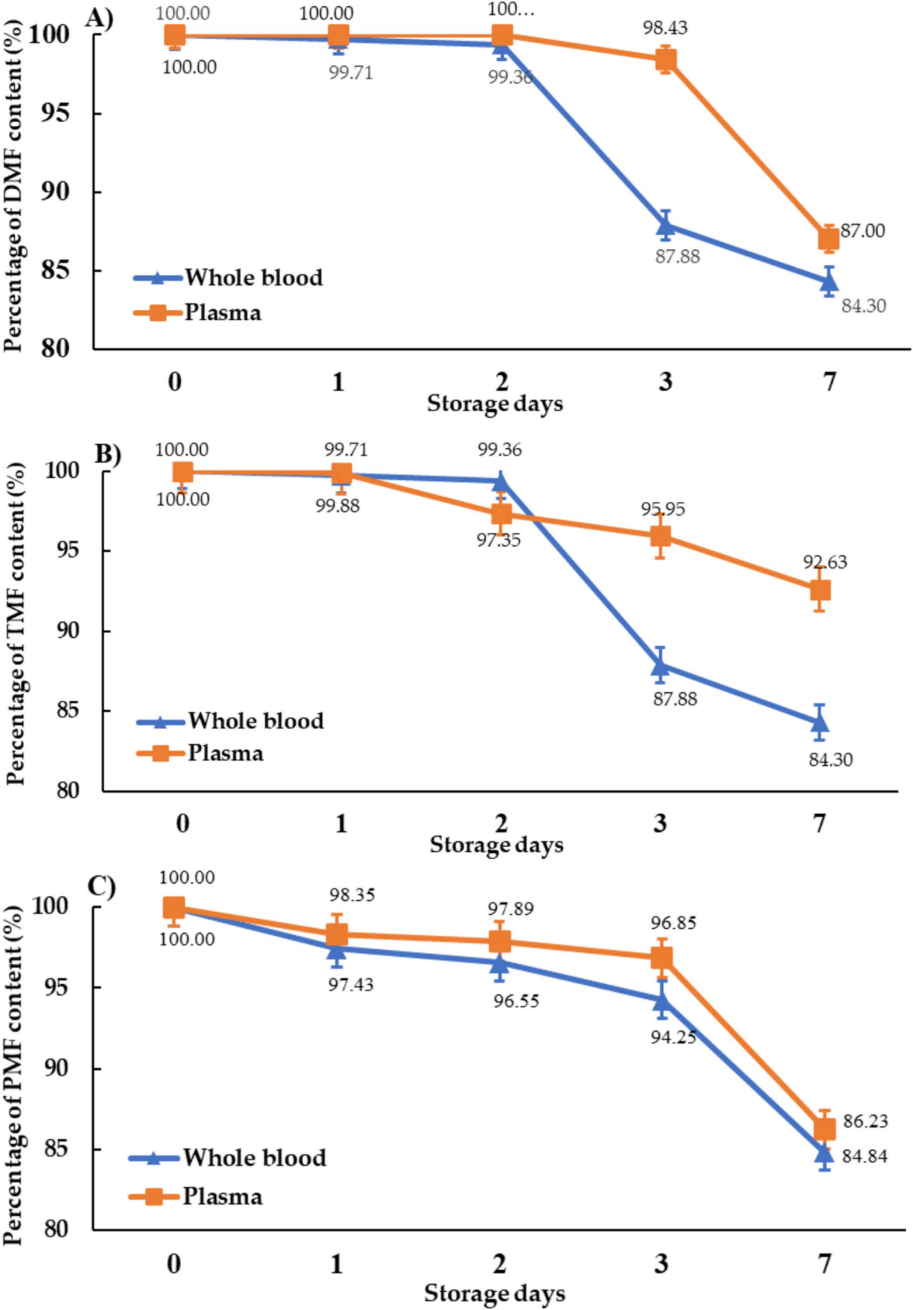
The stability of methoxyflavones for **(A)** DMF, **(B)** TMF, and **(C)** PMF in the blood and plasma of roosters stored at −20°C.

Methoxyflavones remained stable in both blood and plasma stored at −20°C for 2 days (96.6–100% remaining), but significant degradation occurred after 7 days (blood: 84.3–84.6%; plasma: 86.2–92.6%) (Figure 4). This suggests greater stability in plasma compared to whole blood.

## 4 Discussion

This study represents the first pharmacokinetic evaluation of methoxyflavones derived from KP extract in roosters. The results demonstrate a dose-dependent relationship between KP administration and key pharmacokinetic parameters, as demonstrated in Table 4. Increasing the dose from 100 to 200 mg/kg resulted in higher maximum plasma concentrations (C_max_) and greater overall drug exposure (AUC). The given doses of 100, 150, and 200 mg/kg provided C_max_ of methoxyflavones of 0.34–0.64, 0.50–0.81, and 0.54–0.83 μg/mL, respectively. This suggests that the absorption of methoxyflavones in roosters is dose-dependent, highlighting the importance of optimizing dosage regimens for potential applications (22). Methoxyflavones were rapidly absorbed, with peak plasma concentrations reaching 1.17–1.83 h and a relatively slow elimination phase (half-life: 2.03–2.60 h). These findings align with previous research in rats (23) but indicate a longer time to reach peak concentration in roosters, likely due to species-specific differences in digestive physiology (24).

Unlike mammals, birds possess a crop, an expanded portion of the esophagus used for food storage before it enters the stomach. Due to its keratinized lining, the crop has a relatively neutral pH and limited absorptive capacity (25). The pH of the crop is about 6, and some drugs ingested in a solution of drinking water can precipitate in the crop. Therefore, substances passing through the crop may experience delayed transit and absorption. Moreover, the avian stomach consists of two distinct compartments: the proventriculus, responsible for secreting acidic digestive juices, and the muscular gizzard, which grinds food with the aid of ingested grit (25). These differences in digestive physiology likely contribute to the observed variations in absorption kinetics between roosters and rats. Studies have shown that the ileum is the primary site for flavonoid absorption in birds, followed by the jejunum and duodenum (4, 26). The optimal pH for flavonoid absorption is within the range of 5.0–6.8.

Our study highlighted the importance of optimizing extraction methods to accurately quantify methoxyflavones in biological samples. Acetonitrile was a highly effective solvent for extracting methoxyflavones from rooster blood (Table 5). Its efficiency stems from its ability to dissolve these lipophilic compounds, which exhibit low solubility in water, while also causing protein precipitation, thus simplifying sample preparation (27). This finding aligns with previous research demonstrating the efficacy of acetonitrile in extracting methoxyflavones for pharmacokinetic studies (23).

The stability of methoxyflavones in rooster blood and plasma during storage was also assessed (Figure 4). Given that enzymatic activity in plasma can accelerate compound degradation (28, 29), we compared the stability of methoxyflavones in whole blood and plasma stored at −20°C. The results indicated that methoxyflavones concentrations were higher in plasma after 7 days of storage compared to whole blood. This finding suggests that immediate plasma separation following sample collection reduces enzymatic degradation, thereby enhancing the accuracy of subsequent analyses. Previous studies have demonstrated that methoxyflavones in rat plasma degrade over time, even stored at −20°C (19), due to enzymatic metabolism leading to the formation of conjugated derivatives such as O-glucuronides, sulfate esters, and O-methyl esters. The compounds underwent significant first-pass metabolism, partially facilitated by intestinal bacteria, resulting in the degradation into phenolic compounds (30). Our findings confirm the importance of using freshly prepared samples for accurate avian pharmacokinetic studies. The pharmacokinetic data generated from this study is valuable for predicting methoxyflavones blood levels, contributing to the establishment of appropriate dosage regimens and advancing our understanding of lipophilic herbal compound pharmacokinetics in poultry. Moreover, these data suggest KP extract’s potential as a poultry feed additive, particularly during periods of high ambient temperature and relative humidity, such as summer and the rainy season, to mitigate the negative effects of heat stress on poultry productivity. KP extract’s additional anti-inflammatory and antioxidant properties [4, 9, 10] may further enhance poultry immunity, potentially reducing reliance on multiple dietary supplements and lowering production costs.

## 5 Conclusion

In conclusion, this study provides the first report characterizing the pharmacokinetic parameters of a Kaempferia parviflora ethanolic extract in roosters. Our results demonstrate a clear correlation between the administered dose of KP extract and the pharmacokinetic parameters of the three major methoxyflavones (PMF, DMF, and TMF), specifically the maximum concentration (C_max_) and the area under the curve (AUC). Following oral administration, methoxyflavone levels rapidly reached peak concentrations within 1–2 h, followed by slow elimination. These findings are crucial for understanding the absorption, distribution, and elimination of methoxyflavones in roosters. Moreover, our investigation into the stability of methoxyflavones in stored blood samples revealed a significant degradation after prolonged storage, highlighting the importance of prompt sample processing and analysis. Fresh biological samples are strongly recommended to ensure accurate and reliable quantification of methoxyflavones in pharmacokinetic studies. The results provide a comprehensive pharmacokinetic profile of methoxyflavones in roosters and a foundation for their practical application in poultry production.

## Data Availability

The raw data supporting the conclusions of this article will be made available by the authors, without undue reservation.

## References

[1] Vaarst M Steenfeldt S Horsted K. Sustainable development perspectives of poultry production. Worlds Poult Sci J. (2005) 71:609–20. doi: 10.1017/S0043933915002433, PMID: 40109912

[2] Igbokwe NA. Effects of environmental heat stress on reproduction and its management in chickens. Niger Vet J. (2018) 39:101–14. doi: 10.4314/nvj.v39i2.2, PMID: 39807518

[3] Skomorucha I Sosnówka-Czajka E Muchacka R. Effects of supplementing drinking water with mixed herb extract or outdoor access on meat quality characteristics in broiler chickens. Ann Anim Sci. (2020) 20:647–60. doi: 10.2478/aoas-2019-0076

[4] Kamboh AA Leghari RA Khan MA Kaka U Naseer M Sazili AQ. Flavonoids supplementation—an ideal approach to improve quality of poultry products. Worlds Poult Sci J. (2019) 75:115–26. doi: 10.1017/S0043933918000703

[5] Omar JA Hejazi A Badran R. Performance of broilers supplemented with natural herb extract. Open J Anim Sci. (2016) 6:68–74. doi: 10.4236/ojas.2016.61009, PMID: 39241612

[6] Haniarti M Akib MA Ambar A Rusman ADP Abdullah A. Herbal for increasing immunity and weight of poultry. IOP Conf Ser Earth Environ Sci. (2019) 247:012056. doi: 10.1088/1755-1315/247/1/012056

[7] Orczewska-Dudek S Pietras M. The effect of dietary *Camelina sativa* oil or cake in the diets of broiler chickens on growth performance, fatty acid profile, and sensory quality of meat. Animals. (2019) 9:734. doi: 10.3390/ani9100734, PMID: 31569656 PMC6826988

[8] Galli GM Gerbet RR Griss LG Fortuoso BF Petrolli TG Boiago MM. Combination of herbal components (curcumin, carvacrol, thymol, cinnamaldehyde) in broiler chicken feed: impacts on response parameters, performance, fatty acid profiles, meat quality, and control of coccidia and bacteria. Microb Pathog. (2020) 139:103916. doi: 10.1016/j.micpath.2019.103916, PMID: 31812772

[9] Sutthanut K Sripanidkulchai B Yenjai C Jay M. Simultaneous identification and quantitation of 11 flavonoid constituents in *Kaempferia parviflora* by gas chromatography. J Chromatogr. (2007) 1143:227–33. doi: 10.1016/j.chroma.2007.01.033, PMID: 17266972

[10] Sae-wong C Tansakul P Tewtrakul S. Anti-inflammatory mechanism of *Kaempferia parviflora* in murine macrophage cells (RAW 264.7) and in experimental animals. J Ethnopharmacol. (2009) 124:576–80. doi: 10.1016/j.jep.2009.04.059, PMID: 19439175

[11] Wattanapitayakul SK Suwatronnakorn M Chularojmontri L Herunsalee A Niumsakul S Charuchongkolwongse S. *Kaempferia parviflora* ethanolic extract promoted nitric oxide production in human umbilical vein endothelial cells. J Ethnopharmacol. (2007) 110:559–62. doi: 10.1016/j.jep.2006.09.037, PMID: 17113256

[12] Chaturapanich G Chaiyakul S Verawatnapakul V Pholpramool C. Effects of *Kaempferia parviflora* extracts on reproductive parameters and spermatic blood flow in male rats. Reproduction. (2008) 136:515–22. doi: 10.1530/REP-08-0069, PMID: 18614624

[13] Temkitthawon P Hinds TR Beavo JA Viyoch J Suwanborirux K Pongamornkul W. *Kaempferia parviflora*, a plant used in traditional medicine to enhance sexual performance, contains large amounts of low-affinity PDE5 inhibitors. J Ethnopharmacol. (2011) 137:1437–41. doi: 10.1016/j.jep.2011.08.025, PMID: 21884777 PMC4056445

[14] Authaida S Chankitisakul V Ratchamak R Pimpa J Koedkanmak T Boonkum W. The effect of Thai ginger (*Kaempferia parviflora*) extract orally administered on sperm production, semen preservation, and fertility in Thai native chickens under heat stress. Poult Sci. (2023) 103:103372. doi: 10.1016/j.psj.2023.103372, PMID: 38160614 PMC10801310

[15] Cossum PA. Role of the red blood cell in drug metabolism. Biopharm Drug Dispos. (1988) 9:321–36. doi: 10.1002/bod.2510090402, PMID: 3061491

[16] Kaur P Sharma S Choudhury D. Traditional medicine stability and pharmacokinetic issue In: Mandal SC, editors. Evidence based validation of traditional medicines. Singapore: Springer (2021). 677–710.

[17] Bansal G Suthar N Kaur J Jain A. Stability Testing of Herbal Drugs: Challenges, Regulatory Compliance and Perspectives. Phytother Res. (2016) 30:1046–58. doi: 10.1002/ptr.5618 27073177

[18] Tuntiyasawasdikul S Limpongsa E Jaipakdee N Sripanidkulchai B. Transdermal permeation of *Kaempferia parviflora* methoxyflavones from isopropyl myristate-based vehicles. AAPS PharmSciTech. (2014) 15:947–55. doi: 10.1208/s12249-014-0122-y, PMID: 24789664 PMC4113614

[19] Tuntiyasawasdikul S Sripanidkulchai B. LC-MS for determination of three methoxyflavones from *Kaempferia parviflora* in rat plasma and application to pharmacokinetic study. Curr Pharm Anal. (2016) 12:371–8. doi: 10.2174/1573412912666151208212025, PMID: 39962702

[20] Borman P Elder D. Q2(R1) validation of analytical procedures: text and methodology In: Teasdale A Elder D Nims RW, editors. ICH quality guidelines. New York: Wiley (2017). 127–66.

[21] Zhang Y Huo M Zhou J Xie S. PKSolver: an add-in program for pharmacokinetic and pharmacodynamic data analysis in Microsoft excel. Comput Methods Programs Biomed. (2010) 99:306–14. doi: 10.1016/j.cmpb.2010.01.007, PMID: 20176408

[22] Tan TYC Lim XY Krishnan P Muhamad Rosli SH Chan JSW Voon YL. Application of *Kaempferia parviflora*: a perspective review. Nat Prod Commun. (2024) 19:1934578X241281615. doi: 10.1177/1934578X241281615, PMID: 40121490

[23] Mekjaruskul C Jay M Sripanidkulchai B. Pharmacokinetics, bioavailability, tissue distribution, excretion, and metabolite identification of methoxyflavones in *Kaempferia parviflora* extract in rats. Drug Metab Dispos. (2012) 40:2342–53. doi: 10.1124/dmd.112.047142, PMID: 22961680

[24] Houben R Antonissen G Croubels S De Backer P Devreese M. Pharmacokinetics of drugs in avian species and the applications and limitations of dose extrapolation. Vlaams Diergen Tijds. (2016) 85:124–32. doi: 10.21825/vdt.v85i3.16338

[25] Denbow DM. Gastrointestinal anatomy and physiology In: Scanes CG, editor. Sturkie’s avian physiology. 5th ed. New York, NY: Elsevier Inc (2000). 299–325.

[26] Scanes CG Pierzchala-Koziec K. Biology of the gastrointestinal tract in poultry. Avian Biol Res. (2014) 7:193–222. doi: 10.3184/175815514X14162292284822

[27] Oliveira IGC Grecco CF De Souza ID Queiroz MEC. Current chromatographic methods to determine cannabinoids in biological samples: a review of the state-of-the-art on sample preparation techniques. Green Anal Chem. (2024) 11:100161. doi: 10.1016/j.greeac.2024.100161

[28] Aung SH Abeyrathne EDNS Ali M Ahn DU Choi YS Nam KC. Comparison of functional properties of blood plasma collected from black goat and Hanwoo cattle. Food Sci Anim Resour. (2023) 43:46–60. doi: 10.5851/kosfa.2022.e57, PMID: 36789192 PMC9890370

[29] Chen D Li H Li W Feng S Deng D. Kaempferia parviflora and its methoxyflavones: chemistry and biological activities. Evid Based Complement Alternat Med. (2018) 2018:4057456. doi: 10.1155/2018/4057456, PMID: 30643531 PMC6311295

[30] Thilakarathna S Rupasinghe H. Flavonoid bioavailability and attempts for bioavailability enhancement. Nutrients. (2013) 5:3367–87. doi: 10.3390/nu5093367, PMID: 23989753 PMC3798909

